# Non-diabetic elderly populations: SIRI as a risk factor and PIV as a protective factor against bone abnormalities

**DOI:** 10.3389/fendo.2024.1467683

**Published:** 2024-11-14

**Authors:** Manli Yan, Ping Gong, Xiang Li, Haoyue Huang, Hua Wei

**Affiliations:** ^1^ Second Clinical Medical College, Guangzhou University of Traditional Chinese Medicine, Guangzhou, China; ^2^ Department of Orthopedic, Guangdong Provincial Hospital of Chinese Medicine, Guangzhou, China; ^3^ Department of Endocrinology, Guangdong Provincial Hospital of Chinese Medicine, Guangzhou, China; ^4^ State Key Laboratory of Dampness Syndrome of Chinese Medicine, The Second Affiliated Hospital of Guangzhou University of Chinese Medicine, Guangzhou, China

**Keywords:** osteoporosis, elderly populations, non-diabetic populations, SIRI, PIV

## Abstract

**Objective:**

The prevalence of osteoporosis and its resultant healthcare challenges are escalating, posing significant burdens on public health systems. Studies have introduced immunoinflammatory indices, which are recognized for effectively reflecting the systemic immunoinflammatory status. Despite their potential, the exploration of these indices in the context of osteoporosis remains limited. The study sought to explore the relationship between immune inflammation-related indices and osteoporosis in non-diabetic elderly populations.

**Methods:**

The clinical data of 438 non-diabetic elderly subjects were retrospectively analyzed and all statistical analyses were performed using SPSS 27.0.

**Results:**

Differences were observed between the osteoporosis group and the normal bone density group in terms of age, neutrophil, lymphocyte, monocyte, hemoglobin, and platelet. A review of prior studies revealed a close association between osteoporosis and chronic inflammation. Immunological indices such as Platelet to Lymphocyte Ratio (PLR), Neutrophil to Lymphocyte Ratio (NLR), Monocyte to Lymphocyte Ratio (MLR), Systemic Immuno-Inflammatory Index (SII), Systemic Inflammatory Response Index (SIRI) and Peripheral Immunity Index (PIV) were calculated. The analysis indicated significant differences in MLR, SII, SIRI and PIV. A multifactorial binary logistic regression model was established, incorporating age, MLR, SII, SIRI, and PIV as variables. The results identified age and SIRI as independent risk factors for bone abnormalities in non-diabetic elderly populations, while PIV served as an independent protective factor. Receiver operating characteristic analysis demonstrated that SIRI and PIV predicted osteoporosis with areas under the curve (AUC) of 0.609 and 0.620, respectively. The diagnostic value was enhanced when combined with age, yielding AUC values of 0.725 for PIV combined with age. PIV combined with age was particularly effective as a biomarker for bone abnormalities in this population. The optimal Youden’s index was calculated to be 0.367, corresponding to a sensitivity of 63.8% and a specificity of 72.9%.

**Conclusions:**

For non-diabetic elderly populations, SIRI is a risk factor, while PIV serves as a protective factor against bone abnormalities. Combined with previous studies, we suggest that people at high risk of osteoporosis should avoid or minimize the intake of pro-inflammatory dietary patterns. Meanwhile, research from an immune perspective is expected to open new avenues for osteoporosis treatment.

## 1 Introduction

Osteoporosis (OP) is a systemic metabolic bone disorder marked by reduced bone density, increased bone fragility, and the structural degradation of bone tissue. Predominantly associated with aging (1), with a higher prevalence observed in people aged 50 years and older. According to a multicenter cross-sectional study (2), the age-standardized estimated prevalence of osteoporosis in China’s middle-aged and elderly resident population stands at 33.49%, with the prevalence of osteoporosis in people over 50 years of age is 38.05% in females and 20.73% in males; One of the gravest consequences of osteoporosis is hip fracture. Recent global studies indicate (3) that the mortality rate within the first year following a hip fracture ranges between 20-24%. Furthermore, approximately 40% of those affected are unable to walk independently post-fracture, and 60% still require assistance one year later. With the ongoing aging of the population, both the prevalence of osteoporosis and related healthcare costs are projected to escalate (2), presenting significant challenges to the public health systems.

The development of osteoporosis is intricately linked to an imbalance between bone resorption and bone formation throughout the skeletal lifecycle. Research indicates (4, 5) that osteoporosis is strongly correlated with various chronic diseases, where long-term chronic inflammation, such as pancreatitis and rheumatoid arthritis (4, 6), can accelerate the rate of bone loss, thereby reducing bone mineral density (BMD) and leading to a significant increase in fracture risk. During states of inflammation, increased levels of pro-inflammatory cytokines not only enhance osteoclast formation and activity but also impair osteoblast function. This dual effect leads to heightened bone resorption and reduced bone formation, consequently precipitating osteoporosis (7, 8). Additionally, evidence points to elevated levels of C-reactive protein (9) and Interleukin-6 (6) as being closely associated with decreased BMD, suggesting that an inflammatory milieu may intensify bone resorption processes. Oxidative stress and inflammatory responses (10) have been shown to be closely related to bone homeostasis, the mechanisms of which warrant further investigation. A single indicator is not enough to decipher the relationship between disease and inflammation which prompts scholars to delve deeper by analyzing blood cell ratios. The Neutrophil-to-Lymphocyte Ratio (NLR), Platelet-to-Lymphocyte Ratio (PLR) and Monocyte-to-Lymphocyte Ratio (MLR) have been associated with systemic diseases such as diabetes (11), cancer (12) and cardiovascular diseases (13). As research advances, new composite systemic inflammation indices like the Systemic Immune-Inflammation Index (SII), Systemic Inflammation Response Index (SIRI) and Peripheral Blood Immune-Inflammation Index (PIV) have been introduced. These indices, which integrate data from lymphocytes, neutrophils, monocytes and platelets are posited to offer a more comprehensive reflection of the systemic inflammatory status (14).

Reviewing previous studies (15), NLR and SII possess significant predictive capabilities for conditions like postoperative pneumonia in elderly patients with hip fractures. However, the connection between inflammatory immune-related indices and bone-specific diseases like osteoporosis remains underexplored. The current study aims to investigate the correlation between these immune inflammation-related indicators and osteoporosis in non-diabetic elderly populations.

## 2 Information and methods

### 2.1 Objects

Subjects who went to Guangdong Provincial Hospital of Chinese Medicine for physical examination and perfected bone mineral density examination from January 2020 to July 2024 were consecutively included. A total of 438 subjects were recruited as the study subjects after screening for inclusion criteria and exclusion criteria.

#### 2.1.1 Inclusion criteria

① aged ≥50; ② no history of diabetes; ③ complete clinical data.

#### 2.1.2 Exclusion criteria

① Presence of diseases such as infectious states or blood diseases; ② Presence of other endocrine diseases affecting bone metabolism (thyroid diseases, parathyroid diseases, gonadal diseases); ③ Presence of tumor-related diseases such as multiple myeloma; ④ History of radiotherapy or chemotherapy; ⑤ Presence of severe liver and kidney function abnormalities.

#### 2.1.3 Diagnostic criteria for osteoporosis

Referring to the World Health Organisation’s recommended standard, the diagnostic criteria for osteoporosis are as follows: T-value ≥ -1.0 SD measured by dual-energy X-ray absorptiometry is considered normal bone mass, -2.5 SD < T-value < 1.0 SD is considered reduced bone mass, and T-value ≤ -2.5 SD is considered osteoporosis.

### 2.2 Methodology

The study involved a retrospective analysis of dual-energy X-ray BMD and various biochemical indices for 438 participants. All biochemical analyses were conducted at the Guangdong Provincial Hospital of Chinese Medicine, with quality control maintained by the Laboratory Department. The indices assessed included routine blood tests (including neutrophils (NEUT), lymphocytes (LYM), monocytes (MONO), red blood cell (RBC), hemoglobin (Hb), platelets (PLT)), fasting blood glucose (FPG) and glycosylated hemoglobin (HbA1c). NLR is calculated by NEUT/LYM, PLR by PLT/LYM and MLR by MONO/LYM; SII is calculated by PLT × NEUT/LYM, SIRI is calculated by MONO × NEUT/LYM and PIV is calculated by NEUT × MONO × PLT/LYM.

### 2.3 Statistical analyses

The data were analyzed using SPSS 27.0 statistical software, setting α= 0.05 as the threshold for statistical significance; thus, values of p<0.05 were considered statistically significant. The analysis included the use of two independent samples t-tests for normally distributed continuous variables, while non-normally distributed continuous variables were assessed using non-parametric tests, which were expressed as ( $\bar{x}\pm s$ ). Binary logistic regression was employed to explore the correlation between immuno-inflammation-related indices and osteoporosis in non-diabetic elderly populations, which helped to identify which factors are critical influences. Additionally, subject work characteristic (ROC) curves were plotted and the lower part of the curves and the critical values were calculated.

## 3 Results

A total of 438 non-diabetic elderly populations were included, consisting of 216 males and 222 females, with 235 in the osteoporosis group and 203 in the normal bone mass group. Upon comparing general data, differences were observed between the two groups in terms of age, NEUT, LYM, MONO, Hb and PLT (see **Tables 1**, **2**). Previous studies (16) have revealed a close association between osteoporosis and chronic inflammation. Common immune-related indices such as PLR, NLR, MLR, SII, SIRI and PIV were calculated, showing differences in MLR, SII, SIRI and PIV between the groups with statistical significance (see **Table 3**).

**Table 1:** Comparison of general information in non-diabetic elderly populations.

	Osteoporosis group (n=235)	Normal bone mass group (n=203)	P
Age	59.98±6.85	55.47±5.16	<0.001
HbA1c (%)	5.67±0.34	5.64±0.38	0.304
FPG (mmol/L)	5.24±0.54	5.33±0.59	0.326
NEUT (10^9^/L)	3.23±1.20	3.56±1.22	<0.001
LYM (10^9^/L)	1.93±0.57	2.08±0.59	0.012
MONO (10^9^/L)	0.34±0.13	0.40±0.13	<0.001
RBC (10^9^/L)	4.74±0.55	4.86±0.53	0.011
Hb (10^9^/L)	138.99±14.53	144.68±13.92	<0.001
PLT (10^9^/L)	238.91±53.89	254.36±60.47	0.005

**Table 2:** Bone density results between the two groups.

	Osteoporosis group (n=235)	Normal bone mass group (n=203)	P
Lumbar BMD	0.74±0.09	1.11±0.11	P<0.001
Lumbar T-score	-2.98±0.75	0.37±0.94	P<0.001
Hip BMD	2.84±34.21	0.88±0.10	P<0.001
Hip T-score	-2.27±0.61	-0.18±0.74	P<0.001
Femoral neck stress ratio	14.36±3.66	9.16±1.80	P<0.001
Femoral intertrochanteric stress ratio	10.09±1.88	6.81±1.13	P<0.001
Femoral stem stress ratio	3.34±0.97	2.42±0.66	P<0.001

**Table 3:** Comparison of common immune-related indicators between the two groups.

	Osteoporosis group (n=235)	Normal bone mass group (n=203)	P
PLR	133.85±50.24	129.54±39.87	0.720
NLR	1.82±1.00	1.80±0.72	0.113
MLR	0.19±0.11	0.20±0.07	0.015
SII	437.35±262.99	458.80±209.76	0.013
SIRI	0.66±0.74	0.72±0.43	<0.001
PIV	160.61±179.01	187.06±126.52	<0.001

**Figure 1:**
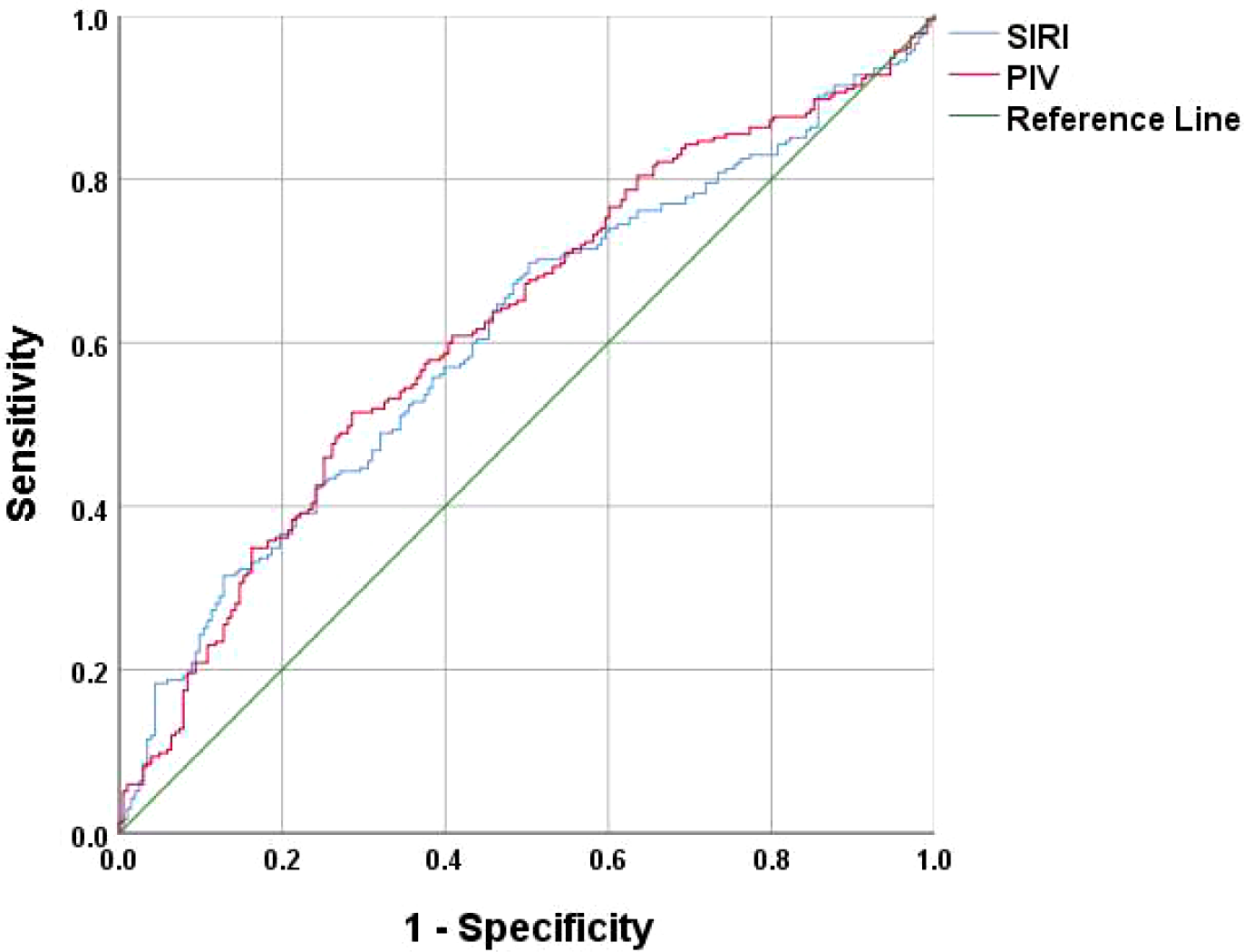
SIRI and PIV as ROC curves for the development of osteoporosis in the non-diabetic elderly populations.

**Figure 2:**
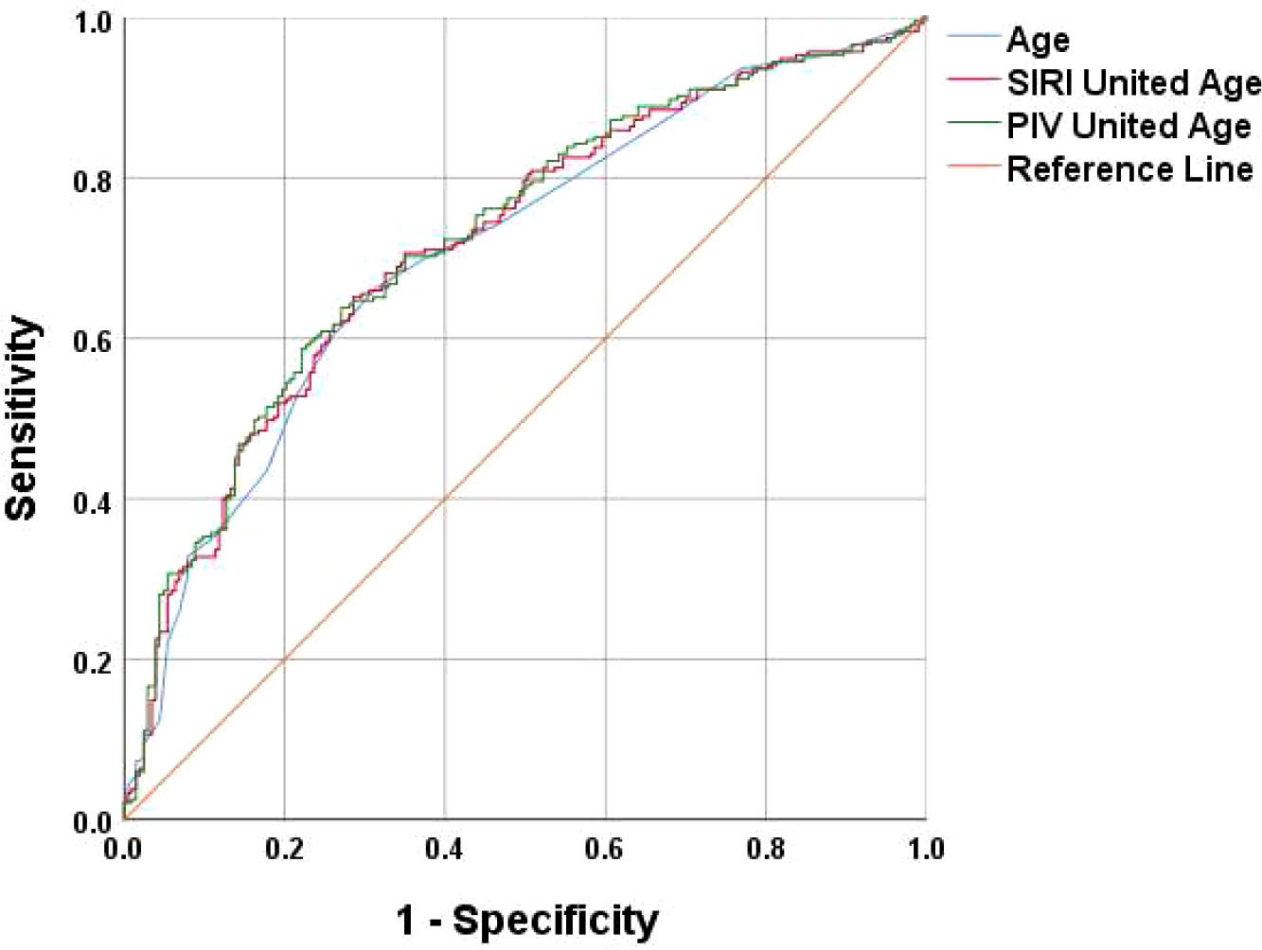
Age, SIRI combined with Age, and PIV combined with Age as ROC curves for the development of osteoporosis in the non-diabetic elderly populations.

A multifactorial binary logistic regression model was established with age, MLR, SII, SIRI and PIV as variables (see **Table 4**). The results indicated that age and SIRI were independent risk factors for abnormal bone density in elderly non-diabetic individuals, while PIV was identified as an independent protective factor. ROC analysis revealed that SIRI and PIV had predictive value for osteoporosis in non-diabetic elderly individuals, with areas under the curve of 0.609 and 0.620, respectively (see **Figure 1**). Age, age combined with SIRI and age combined with PIV showed higher diagnostic values for abnormal bone density in non-diabetic elderly individuals, with area under the curve of 0.709, 0.720 and 0.725. PIV combined with age was deemed more suitable as a marker for abnormal bone density in non-diabetic elderly individuals. The optimal PIV combined with the age cut-off value was determined using Youden’s index. The cutoff value with the highest Youden index (0.367) was defined as optimization, with sensitivity and specificity of 63.8% and 72.9%, respectively (see **Figure 2**).

**Table 4:** Independent predictors of the occurrence of osteoporosis in the non-diabetic elderly population analyzed by binary logistic regression.

	B	Wald X2	OR	95%CL	P
Age	0.141	49.465	1.151	1.107~1.198	<0.001
MLR	-3.085	1.618	0.046	0.000~5.306	0.203
SII	0.001	2.403	1.001	1.000~1.003	0.121
SIRI	1.730	5.682	5.640	1.360~23.388	0.017
PIV	-0.009	8.574	0.991	0.986~0.997	0.003

## 4 Discussion

Osteoblasts, osteoclasts, and immune cells have long been recognized to communicate through shared signaling mechanisms. Recent studies have revealed a close relationship between bone health and immune response, with some scholars even proposing the academic viewpoints of “osteoimmunology” and “immune osteoporosis” (16).

It is generally believed that an imbalance in bone metabolism, where bone resorption by osteoclasts exceeds bone formation by osteoblasts, leads to osteoporosis. Aging (17) is considered a risk factor for osteoporosis in the elderly because aging results in a decrease in the number and differentiation capacity of marrow stromal stem cells (MSCs), which are the main source of osteoblasts, leading to reduced bone formation. Platelets play a supportive role in bone formation. Studies (18) have shown that platelet derivatives (leukocytes and platelet-rich fibrin) enhance the healing of ovariectomy-induced Xenotransplantation in osteoporotic rats.

Moreover, hormonal changes and chronic inflammation due to aging significantly contribute to the abnormal activation of osteoclasts. Osteoclasts (19) are essentially multinucleated giant cells with bone resorptive functions, whose formation is regulated by various blood cells. For instance, NEUT (20) can express the osteoclast-activating factor RANKL, while LYM (16) can secrete granulocyte colony-stimulating factor (G-CSF). Importantly, these reactions are not entirely independent. Research (21) has also shown that the addition of signaling factors like RANKL and M-CSF to culture dishes containing MOMO can promote the fusion of monocytes into multinucleated giant cells, ultimately leading to osteoclast formation. Additionally, a decrease in Hb (22) can stimulate the proliferation of hematopoietic cells, including osteoclasts, potentially increasing bone resorption. Although blood loss can also stimulate osteoblast formation, excessive bone resorption may impede the bone remodeling cycle, resulting in osteoblast fatigue. The data from this study indicate that subjects in the osteoporosis group were older, with lower levels of NEUT, LYM, MOMO, Hb and PLT compared to the normal bone mass group, and these differences were statistically significant.

As research progresses, it has become evident that the relationship between bone marrow cells and osteoporosis is not a simple linear one. Studies (16, 23) have shown that LYM plays a crucial regulatory role in the bone remodeling process. Activated T-lymphocytes can enhance bone resorption and lead to bone loss by secreting RANK (24), various inflammatory factors, and related ligands such as TNF-α (25), IL-1 (26) and IL-6 (26). Conversely, resting T-lymphocytes (23) can protect osteoclasts from bone resorption. Mature B-lymphocytes (27) can activate BTK tyrosine residues through receptor signaling, which plays a critical role in osteoclast differentiation. However, the maturation of B lymphocytes heavily relies on secretory factors from matrix cells and osteoblasts (16), allowing for a delicate balance between osteoblasts and osteoclasts under physiological conditions.

Nevertheless, this balance can be disrupted in chronic inflammatory states, promoting some researchers to propose the use of immunotherapy for treating osteoporosis (28), such as T cell therapy.

In conclusion, the relationship between immune cells and osteoporosis is complex. It has been observed that a single indicator is insufficient to explain the connection with osteoporosis fully. Some researchers have started exploring the relationship between different subgroups and osteoporosis by calculating immune-inflammatory ratios (PLR, NLR, MLR). Gao,K et al. (29)conducted a cross-sectional study involving 181 osteoporosis patients and 111 healthy subjects, suggesting early on that MLR holds significant diagnostic value for osteoporosis. However, this study did not specifically mention the endocrine status of the subjects included or the correlation between MLR and BMD or stress ratio. Following research by Li H et al. (30), it was concluded that MLR could serve as an independent protective factor for postmenopausal T2DM patients with osteoporosis, exhibiting a positive correlation with lumbar spine BMD and hip BMD. The results of this study also indicate differences in MLR between the osteoporosis and normal bone mass groups in elderly non-diabetic individuals.

Recently, scholars have proposed the concept of comprehensive systemic immune-inflammatory indicators, with SII, SIRI, and PIV being common novel immune-inflammatory biomarkers involving various peripheral blood immune cell subgroups - NEUT, PLT, MOMO and LYM. These indicators are considered more comprehensive in representing a patient’s immune status and systemic inflammation. Studies on SII, SIRI, PIV and similar markers have been widely applied in research related to the occurrence and development of tumors (31), but their use in orthopedic research has been relatively limited.

ZHANG J X et al. (32)conducted a cross-sectional study involving women aged 20 and above (n=4092) by collecting data from the National Health and Nutrition Examination Survey (NHANES) from 2007 to 2010. Their findings indicated a negative correlation between SII levels in postmenopausal women and BMD levels, while no association was observed between SII levels and BMD in premenopausal women. Elevated SII levels may serve as a potential risk factor for osteoporosis in postmenopausal women; TANG YC et al. (33) analyzed postmenopausal women aged 50 and above (n=893) by collecting data from NHANES from 2007 to 2018. Their study suggested that SII could be used to predict the risk of low BMD or osteoporosis in postmenopausal women aged 50 and above. Data from this study indicated a difference in SII between the osteoporosis and normal bone mass groups (P=0.013), but binary logistic regression analysis found it to be statistically insignificant.

Ma, H. et al. (34) retrospectively analyzed elderly hypertensive patients (age ≥60 years, n=856) who visited the People’s Hospital of Xinjiang Uygur Autonomous Region from January 2021 to December 2023. Their research revealed that with increasing SIRI, the risk of osteoporosis increased, and a potential association existed between SIRI and BMD, osteoporosis, and the future risk of fractures in elderly hypertensive patients. The data analysis from this study also identified SIRI as a risk factor for osteoporosis in non-diabetic elderly individuals(OR=5.640), consistent with previous research findings.

Wang, X. et al. (35) calculated the Dietary Inflammatory Index (DII) using a statistical diet frequency questionnaire. The results of the retrospective analysis indicated a significant positive correlation between the DII and the SIRI. The study emphasized the association between dietary inflammation and blood inflammation, suggesting that the two have a strong synergistic effect. The Mediterranean diet has been demonstrated to reduce the DII index in previous studies (36), and a study by Wang, Q (37). has shown that different dietary patterns can influence DII. Therefore, a reduction in pro-inflammatory dietary patterns is recommended for individuals at high risk of developing osteoporosis. Currently, there is a lack of research on PIV and osteoporosis. This study concludes that PIV is an independent protective factor for abnormal bone density in elderly non-diabetic patients. Frailty is considered one of the common health problems in the elderly, similar to osteoporosis. In frail populations (38) excessive immune-inflammatory responses can result in significant health risks and may serve as a potential underlying cause of sarcopenia associated with frailty. In contrast, elderly individuals who have not yet displayed characteristics of frailty (i.e., pre-frail participants) may be at an increased risk of mortality if their levels of NLR, MLR, SIRI and PIV are insufficiently low. Some researchers suggest that (39) immune senescence may be a critical factor contributing to the decline in bodily function. Cui, Y (40). et al. conducted a study utilizing bone-targeted biomimetic nanogel technology to restore the balance between osteoblasts and osteoclasts for the treatment of postmenopausal osteoporosis. This approach demonstrated favorable therapeutic outcomes in a mouse model. Consequently, exploring the immune-related mechanisms underlying osteoporosis may offer new therapeutic strategies, such as developing drugs or nutritional supplements targeting inflammatory mediators, which could potentially alleviate the symptoms of osteoporosis.

Since BMD technology is not fully popular, the diagnosis of osteoporosis is difficult in some areas. Both SIRI and PIV are derived from routine tests and do not require special techniques, potentially offering clinicians a novel, cost-effective, and readily accessible method for predicting osteoporosis. Further in-depth research on target mechanisms is needed.

In this study, we preliminarily explored the potential application of SIRI and PIV in the diagnosis of osteoporosis. However, the study has certain limitations, such as a small sample size and a relatively limited patient population, which may affect the generalizability of the results. Additionally, as this is a retrospective analysis, there may be potential selection bias. In response to these limitations, we have considered future research directions, such as increasing the sample size and study population, as well as conducting more rigorous multicenter prospective cohort studies to reduce bias and enhance the accuracy and stability of the findings. Moreover, we will further investigate the specific biological mechanisms between SIRI, PIV and bone metabolism. Particularly the underlying mechanisms of bone formation and resorption balance in different inflammatory environments, to provide stronger evidence to support future clinical diagnostics.
